# A role for CD81 on the late steps of HIV-1 replication in a chronically infected T cell line

**DOI:** 10.1186/1742-4690-6-28

**Published:** 2009-03-11

**Authors:** Boyan Grigorov, Valérie Attuil-Audenis, Fabien Perugi, Martine Nedelec, Sarah Watson, Claudine Pique, Jean-Luc Darlix, Hélène Conjeaud, Delphine Muriaux

**Affiliations:** 1 LaboRetro, Unité de virologie humaine INSERM U758, Ecole Normale Supérieure de Lyon, IFR128, 46 allée d'Italie, 69364 Lyon, France; 2 Institut Cochin, Département de Biologie Cellulaire, CNRS 8104, INSERM 567, Paris V, 22 rue Méchain, 75014 Paris, France

## Abstract

**Background:**

HIV-1 uses cellular co-factors for virion formation and release. The virus is able to incorporate into the viral particles host cellular proteins, such as tetraspanins which could serve to facilitate HIV-1 egress. Here, we investigated the implication of several tetraspanins on HIV-1 formation and release in chronically infected T-lymphoblastic cells, a model that permits the study of the late steps of HIV-1 replication.

**Results:**

Our data revealed that HIV-1 Gag and Env structural proteins co-localized with tetraspanins in the form of clusters. Co-immunoprecipitation experiments showed that Gag proteins interact, directly or indirectly, with CD81, and less with CD82, in tetraspanin-enriched microdomains composed of CD81/CD82/CD63. In addition, when HIV-1 producing cells were treated with anti-CD81 antibodies, or upon CD81 silencing by RNA interference, HIV-1 release was significantly impaired, and its infectivity was modulated. Finally, CD81 downregulation resulted in Gag redistribution at the cell surface.

**Conclusion:**

Our findings not only extend the notion that HIV-1 assembly can occur on tetraspanin-enriched microdomains in T cells, but also highlight a critical role for the tetraspanin CD81 on the late steps of HIV replication.

## Background

Tetraspanins constitute a large family of membrane glycoproteins with four transmembrane domains which are widely expressed in human cells. The tetraspanin family comprises 33 different members, among which the most studied are CD9, CD63, CD81, CD82 and CD151. These proteins have a role in the regulation of many biological processes such as cell-cell adhesion, fusion, signal transduction, proliferation and differentiation [1,2]. The exact mechanism by which these proteins function is still poorly understood. Tetraspanins probably function in the form of complexes since they interact with each other and with different partners including transmembrane proteins such as adhesion molecules, receptors and intracellular signalling/cytoskeletal proteins, creating a network of interacting proteins called the tetraspanin web [3]. Their ability to also interact with cholesterol has led to the concept that tetraspanins might be organizers of specific lipid microdomains which are referred to as tetraspanin-enriched microdomains (TEMs) [4-6]. Tetraspanins also play a role in the dissemination of pathogens that cause malaria and diphtheria and in viral infections [7]. Moreover, several tetraspanins are involved in the life cycle of certain viruses, beginning from their initial cellular attachment and ending with virus production. In this respect, CD81 is probably the best known example in its role as a binding partner of the E2 envelope protein of HCV [8,9].

Recent investigations have focused on the involvement of tetraspanins in human immunodeficiency virus type 1 (HIV-1) assembly. In fact, HIV-1 assembly has been shown to take place mainly at the plasma membrane, but also in multivesicular body (MVB)/late endosomes [10-20], even though this latter location for HIV-1 has been recently challenged by investigators who reported that the endosomal HIV-1-containing compartments in macrophages could actually be deep invaginations of the plasma membrane [21,22]. Nevertheless, it remains that HIV-1 assembly seems to favour tetraspanin-enriched microdomains (TEMs) [12,21,16,23]. Tetraspanins can be found at the cell surface and in intracellular compartments: CD63, which possesses an interacting motif with the adaptor AP-3 protein, is mainly targeted to the endocytic pathway [24] while most of the other tetraspanins are found both at the plasma membrane and in intracellular vesicles [25]. Indeed, late endosomes/MVBs are highly enriched in the tetraspanins CD9, CD63, CD81, and CD82, which contribute to their fusion with the plasma membrane and the release of 50–90 nm vesicles called exosomes that resemble viral particles [26,25,27].

If HIV-1 assembly takes place on tetraspanin-enriched microdomains (TEMs), proteins from these domains would be expected to be incorporated during virus formation into newly made virions. In agreement with this notion, HIV-1 budding structures and newly made HIV-1 particles can be labeled by anti-CD63 antibodies, as shown by immuno-electron microscopy [14,28,29]. We previously reported the association of CD63 with HIV-1 particles and HIV-1-containing compartments in an infected T-lymphoblastic cell line [14]. In addition, CD63, found mainly in MVBs, is incorporated into HIV-1 virions [12,14,20,30]. Yet, recent works have reported a contradictory role of CD63 on the late steps of HIV replication in macrophages [31,32].

It was thus proposed that HIV-1 exploits the exocytic vesicular pathway for its assembly and budding. However, CD81 was also found to co-localize with HIV-1 Gag protein at the surface of Jurkat T cells and in exosomes [12], as well as with HIV-1 virions accumulated in CD81 and CD9 enriched intracellular compartments of dendritic cells [33]. Finally, a recent report showed that CD63 and CD81 are recruited within the virological synapse and contributed to the formation of this structure [16]. These findings indicate that CD63, CD81 and possibly other tetraspanins can be involved in HIV-1 assembly, but their precise role in HIV-1 biogenesis remains to be determined.

To address this question, we investigated the relationships between Gag, which is the major structural polyprotein of HIV-1, and several tetraspanins such as CD9, CD63, CD81 and CD82 in chronically infected T lymphoblastic cells (MOLT/HIV-1 cells). This cell line appears to be a good model to study the last steps of the virus life cycle because the expression of CD4, the HIV-1 receptor, is downregulated below detectable level; thus, this lack of CD4 should prevent reinfection of the cells. We have previously reported in MOLT/HIV-1 cells a phenotype atypical of HIV-1 infected T cells in which there is a high level of late endosome-associated viral particles [14]. By means of confocal microscopy imaging, viral and cellular biology technics, we report that in the MOLT/HIV-1 cell line, there is a clustering of the tetraspanins CD63, CD81 and CD82 together with the viral structural proteins Gag and Env. In addition, the latter tetraspanins co-purified with HIV-1 virions. However, not all of the tetraspanins seem to have a critical role in HIV-1 formation since our results showed that intracellular Gag protein was part of protein complexes containing mainly CD81 and much less CD82, suggesting a possible major role of CD81 in virus assembly. As a consequence, CD81 and limited CD82 were incorporated in purified HIV-1 virions. Finally, when HIV-1 producing cells were treated with anti-CD81 antibodies, or when CD81 was downregulated by RNA interference, HIV-1 production was impaired and Gag became evenly distributed at the cell surface.

Our results show that HIV-1 assembly can occur on tetraspanin-enriched microdomains in MOLT/HIV-1 cells and that the tetraspanin CD81 recruited in the viral particles plays a critical role in the late steps of HIV-1 replication.

## Materials and methods

### Cell culture

Chronically HIV-1_NL4-3 _infected MOLT lymphocytes were used in this study and were a kind gift of J. Esté (University of Barcelona, Spain). Parental MOLT-4 cells are prototype lymphoid T cells (NIH AIDS reagent program, USA) and were infected with HIV-1_NL4-3 _to generate chronically infected MOLT cells. These cells are negative for CD4 as measured by flow cytometry. MOLT/HIV-1 and SupT1 (T-lymphocitic cell line) cells were grown in RPMI supplemented with 10% fetal calf serum (FCS) and antibiotics.

### Antibodies

Immunoblotting, immunostaining, immunoprecipitations and cell surface tetraspanin "depletion" were performed using the following antibodies: rabbit anti-MAp17, mouse anti-CAp24, mouse anti-TMgp41, human anti-SU gp120 (NIH, USA), the mouse monoclonal antibodies anti-Lamp2 (H5G11), anti-Lamp3/CD63 (MX-49.129.5), anti-CD81 (5A6), anti-GAPDH (6C5), (Santa Cruz Biotechnology Inc.), anti-CD45 (HI30) (BD Pharmingen). Anti-CD9 (Syb1), anti-CD63 (TS63) and anti-CD81 (TS81) were mouse IgG1 antibodies from ascitic fluids, and were kind gifts from E. Rubinstein. Anti-CD82 (alphaC11) was a purified mouse IgG1 antibody (2 mg/ml). Anti-VsV-g (P5D4) was used as an irrelevant antibody. For immunofluorencence staining, fluorescent Alexa^® ^488, 546 and 633-conjugated secondary antibodies were used (Molecular Probes).

### Immunofluorescence staining and confocal microscopy imaging

MOLT/HIV-1 cells were harvested by centrifugation, washed once in PBS, and fixed in 3% paraformaldehyde-PBS for 20 minutes. The fixative was then removed, and free aldehydes were quenched with 50 mM NH_4_Cl. The necessary cells were then permeabilized using 0.2% Triton X-100 for 5 minutes and blocked in 1% BSA-PBS. The fixed cells were incubated for one hour at room temperature with primary antibodies, washed 3 times with 1% BSA-PBS, and further incubated for 1 hour with the corresponding secondary fluorescent antibodies. The slides were mounted with Mowiol (Sigma). Images were acquired on Axioplan 2 Zeiss CLSM 510 confocal microscope with Argon 488/458, HeNe 543, HeNe 633 lasers and plan apochromat 63 × 1.4 oil objective, supplied with LSM 510 3.4 software. Co-localization between Gag and cellular markers was determined using the MetaMorph^® ^OffLine 7.0 Software.

### Virion purification and immunoblotting

HIV-1 virions produced by MOLT/HIV-1 cells were purified by pelleting through a double layer of 25%–45% sucrose cushion in TNE (Tris 10 mM, NaCl 100 mM, EDTA 1 mM) at 28,000 rpm for 1 hour and 15 minutes in a SW28 Beckman rotor. The 5 ml sucrose-cushion interphases containing the virions were collected and diluted in PBS. The virions were further purified by another ultracentrifugation through a 25% sucrose-TNE cushion, and resuspended in TNE.

Viral pellets or cell lysates (50 μg of total cellular proteins per lane) were separated on 10% SDS-PAGE and detected by immunoblotting with primary and secondary antibodies as follows: mouse anti-CAp24, mouse anti-Lamp2, anti-Lamp3, anti-CD81, anti-CD9, anti-CD82 and anti-CD45. Corresponding horse-radish-peroxidase (HRP) conjugated immunoglobulins (DakoCytomation) were used and the signal was detected using SuperSignal^® ^West Pico Chemiluminescent Substrate (Pierce).

### Sucrose gradient fractionation

Viral pellets from MOLT/HIV-1 were resuspended and layered on top of a discontinuous sucrose gradient (20–60%) and ultracentrifuged for 18 hours at 25,000 rpm in SW41 rotor. 500 μl fractions were collected and measured for density using a refractometer. All fractions were analysed for the presence of virus particles both by exogenous reverse transcriptase (RT) activity and by immunoblotting with anti-CAp24 and anti-TMgp41 antibodies. The fractions were analyzed by immunoblotting and for the presence of tetraspanins and other cellular proteins as already described.

### Reverse Transcriptase assay

RT activity of viral immunoprecipitated supernatant was measured using the following procedure: 12 μl of virus containing supernatant were incubated in a mix containing 48.75 μl of RT buffer (60 mM Tris pH 8.0, 180 mM KCl, 6 mM MgCl_2_, 0.6 mM EGTA pH 8.0, 0.12% Triton X100), 0.3 μl of 1 M DTT, 0.16 μl of 2 mg/ml oligo dT, 0.6 μl of 1 mg/ml poly rA and 0.25 μl of alpha^32^P dTT for 1 h at 37°C. Afterwards, 5 μl of this reaction were deposited on a Whattman paper, and the latter was quickly washed twice with 2 × SSC (0.3 M NaCl, 0.03 M sodium citrate pH 7.0) and then once more for 15 minutes. The membrane was exposed on a phosphor screen and read using a phosphorimager.

### Immunoprecipitation of HIV-1 virions

For each virus immunoprecipitation experiment, the appropriate quantity of antibody (2 μg for anti-CD9, CD63, CD81 and CD82, 1.5 μg for anti-CD45 and anti-tubulin and 3 μl for human anti-HIV serum) was mixed with 50 μl of Protein G Sepharose™ beads, and incubated for 1 hour on ice. Purified HIV-1_NL4-3 _virions (10^7 ^virions/sample) produced by chronically infected MOLT cells were diluted in 200 μl of 0.1% BSA-PBS and were mixed with 12 μl of Protein G Sepharose™ beads and incubated for 1 hour at 4°C on a wheel. After centrifugation, the precleared virions were added to Protein G Sepharose™ beads coupled with different antibodies and incubated for 4 hours at 4°C on a wheel. Then after centrifugation, the virion-coupled-beads were washed 3 times in PBS and resuspended in reducing electrophoresis buffer for a SDS-PAGE analysis. Immunoprecipitated virus was detected by immunoblotting using an anti-CAp24 (rabbit, from Aids Reagent Program) antibody and an anti-mouse HRP-conjugated antibody.

### Intracellular immunoprecipitation

Proteins from 4.10^7 cells were solubilized in 1 ml TBS-CHAPS (Tris/HCl 50 mM + 150 mM NaCl + 1 mM CaCl2 + 1 mM MgCl2, pH 7.4), supplemented with 1% CHAPS and protease inhibitors. After a preclearing step of 2 hours incubation with 50 μl Protein G Agorose beads (Roche Diagnostic), cell lysates were incubated overnight at 4°C with 2 μg of immunoprecipating antibodies (anti-CD82, anti-CD81, anti-CD63, anti-CD9 or control antibody (anti-GAPDH or anti-CD71) and 2 hours with Protein G-Agarose beads (50 μl/IP). Immunoprecipitated materials (obtained after 5 washes in TBS-CHAPS) were solubilized in 50 μl of 1× sample buffer without reducing agent, while supernatants were diluted 10 times in 1.1 sample buffer. After 10 minutes boiling, proteins were separated by 12% SDS-PAGE and transfered on PVDF membranes. The membranes were incubated with blocking buffer TBST (TBS 1× + 0.1% Tween 20) + 5% non-fat milk and then with mouse anti-CD82 or rabbit anti-CAp24. After 5 washes in TBST supplemented with 1% skim milk, membranes were incubated with HRP-secondary antibodies (anti-mouse or anti-rabbit), washed extensively (5 times) and probed with ECL+ Western blotting detection kit (Amersham).

### FACS analysis

For cell surface analysis, MOLT/HIV-1 cells were incubated for 30 minutes at 4°C with a saturating concentration of antibodies directed against CD81 and CD45 in PBS 5% FCS. An irrelevant antibody was also used as a staining control. After 2 washes in PBS 5% FCS, cells were fixed in PBS 3% PFA for 10 minutes and washed once. Then goat anti mouse -Phyco-erythrine (PE) labeled (GAM-PE) was used as a secondary antibody (Santa Cruz Biotechnology Inc). For overall staining, cells were fixed, stained and permeabilized with the Fix and Perm^® ^cell permeabilization reagents, according to manufacturer's instructions, then GAM-PE was used as a secondary antibody. For Fig. SixB, directly PE-conjugated antibodies against CD81 (JS-81) or CD45 (HI30) from BD-Pharmagen were used at a saturating concentration. Data acquisition and analysis were performed with FACS calibur flow cytometer equipped with CellQuest Pro software (BD Biosciences).

### CD81 silencing using lentivectors and infectivity

Lentivectors were produced as follow: 293T cells were transfected with the plasmids expressing the envelope VSV-G, the HIV-1 Gag-Pol [34] and the shRNA directed against CD81 (UCAUGAUGUUCGUUGGCU) or the control shRNA (GACCCCCUUGTGAAUCUC). GFP was included as a marker in the lentivector (a kind gift of Birke Bartosch, Inserm#758, ENS de Lyon, France). Vectors are derived from [35]. Lentivector particles were collected 48 hours post-transfection and purified by ultracentrifugation on a 25% sucrose cushion. The virus titer was determined by measuring GFP expression in HeLa cells by FACS analysis. The same amount of VLPs was used to transduce MOLT/HIV-1 cells at a multiplicity of infection (MOI) of 2 for 72 hours. Then cells were washed in PBS and resuspended in a new medium for 6 hours to let *de novo *release of HIV-1. Fifteen μl of HIV-1 containing supernatant were collected for an RT test, and the rest was purified by spinning at 2000 rpm/5 min and ultracentrifuged afterwards at 50 000 rpm/2 h/4°C in a TL-100 rotor and analysed by immunoblotting. One part of the cells was analyzed by flow cytometry (for GFP expression to monitor transduction efficiency, and to monitor for CD81 downregulation). The remaining cells were lysed in RIPA, sonicated and 70 μg of total protein were analysed by immunoblotting.

For infectivity measurement, 10 μl of each virus pellet, produced by shRNA treated cells, were added to SupT1 cells and incubated overnight. Afterwards cells were washed using PBS and were resuspended in new medium for 6 days. When syncytia formation occurred, the cell supernatants were collected and *de novo *virus production was measured to determine infectivity by exogenous RT activity. Results were normalized after taking into account of the initial RT activity of the viral innoculum.

## Results

### Localization of tetraspanins and viral proteins in HIV-1 infected T cells

We analyzed by immuno-confocal microscopy the influence of HIV-1 infection of MOLT T lymphoblastic cells (MOLT/HIV-1) on the cellular distribution of several tetraspanins, such as CD9, CD63, CD81 and CD82, in comparison with other membrane proteins, such as the CD45 tyrosine phosphatase, which is known as a marker of the plasma membrane [36], and Lamp2 which is a lysosome-associated membrane protein [37].

**Figure 1:**
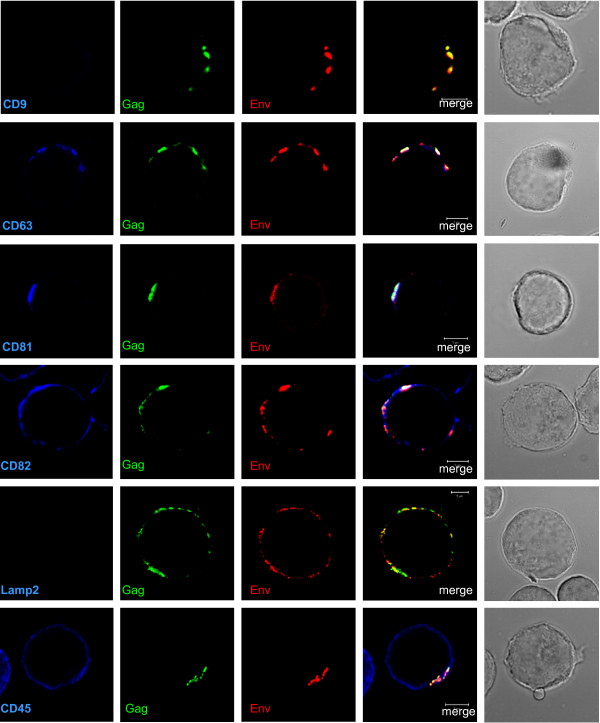
**Localization of HIV-1 Gag and Env with tetraspanins at the cell surface of HIV-1 infected MOLT cells**. MOLT/HIV-1 cells were fixed and the cell surface was stained directly with the anti-tetraspanin CD9, CD63, CD81 or CD82 antibodies, or with antibodies against CD45 or Lamp2. To reveal the viral proteins Gag and Env, the cells were co-stained with anti-MAp17 (Gag in green) and anti-SU gp120 (Env in red) antibodies. It can be observed that the tetraspanins are localized in microdomains close to or at the cell periphery. The percentage of Gag co-localization with the markers was calculated by image analysis and reported in the graph (Fig. 3).

In a first series of experiments, MOLT/HIV-1 cells were surface labelled with specific antibodies against viral and cellular proteins without permeabilization of the cell membrane (Fig. 1). At the cell surface of MOLT/HIV-1 cells, CD81 co-localized with both Gag and Env in discrete plasma membrane clusters while CD63 and CD82 mainly co-localized with Gag and partially with both Gag and Env (Fig. 1, merge in white color). CD45 remained evenly distributed at the cell surface and only sometimes co-localized with Gag and Env. In contrast, both CD9 and Lamp2 were undetectable (Fig. 1). One could notice that in the absence of permeabilization, some Gag protein was found at the cell surface. This might be explained by the fact that cell fixation by PFA can partially permeabilized cells or viruses at the cell surface rendering Gag accessible to the antibody even if Gag molecules are within the virions that are departing the cell as shown by electron microscopy [14].

**Figure 2:**
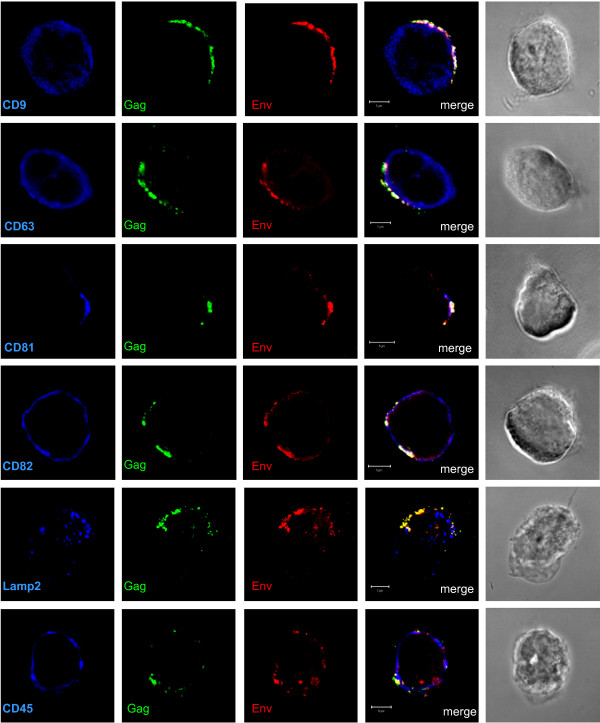
**Localization of HIV-1 Gag and Env with tetraspanins in permeabilized HIV-1 infected MOLT cells**. MOLT/HIV-1 cells were fixed, permeabilized, and stained with the anti-tetraspanin CD9, CD63, CD81 or CD82 antibodies, or with antibodies against CD45 or Lamp2. To reveal the viral proteins Gag and Env, the cells were co-stained with anti-MAp17 (Gag in green) and anti-SU gp120 (Env in red) antibodies. The percentage of Gag co-localization with the markers was calculated by image analysis and reported in the graph (Fig. 3).

In a second series of experiments, we compared the overall distribution after cell permeabilization of the same cellular proteins and HIV-1 Gag and Env (Fig. 2). Unlike the surface staining, permeabilization showed that Gag and Env fully co-localized with CD63, CD81 and CD82. Surprisingly, CD9, which was not detected at the cell surface (Fig. 1), was found in intracellular compartments and co-localized with HIV-1 Gag and Env in clusters near the plasma membrane. No co-localization was observed with Lamp2, suggesting that the tetraspanin/HIV-1 enriched intracellular compartments did not correspond to lysosomes. A partial co-localization of Gag and Env appeared with the CD45 plasma membrane protein.

**Figure 3:**
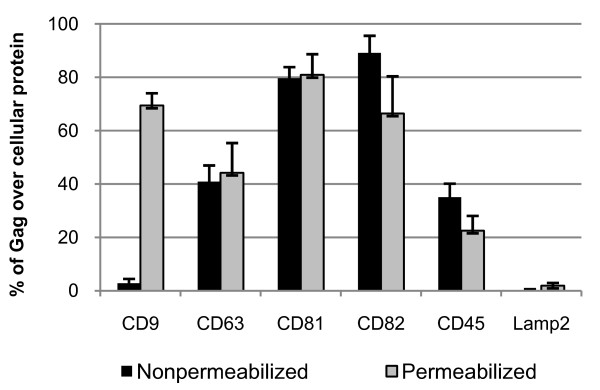
**Localization of HIV-1 Gag and Env with tetraspanins in HIV-1 infected MOLT cells**. The percentage of Gag co-localization with the tetraspanins or the CD45 or Lamp2 proteins was calculated by image analysis by the MetaMorph^® ^Software and reported in the graph. Quantifications in non permeabilized MOLT/HIV-1 cells are indicated in black color, and in permeabilized cells in grey color, as indicated.

Quantification of Gag co-localization with tetraspanins revealed that Gag was mainly distributed within CD81 and CD82 labelled microdomains for non-permeabilized cells (i.e. 80% of Gag co-localized with CD81 and CD82, and 40% and 30% with CD63 and CD45, respectively), and within CD9, CD81 and CD82 labelled microdomains upon cell permeabilization (i.e. between 40–80% of Gag co-localized with CD9, CD63, CD81 and CD82 tetraspanins, and only 20% with the CD45 cell surface protein) (Fig. 3).

Altogether, these results indicate that Gag and Env co-localized mainly with the tetraspanins CD81, CD82 and less often with CD63 in membrane microdomains (called TEM complexes) or near the cell surface, but very little with other membrane proteins like Lamp 2 and CD45.

### Co-fractionation of Purified HIV-1 virions with tetraspanins

To explore the functional relationship between HIV-1 assembly and TEMs, we investigated the possible incorporation of tetraspanins into progeny virions.

**Figure 4:**
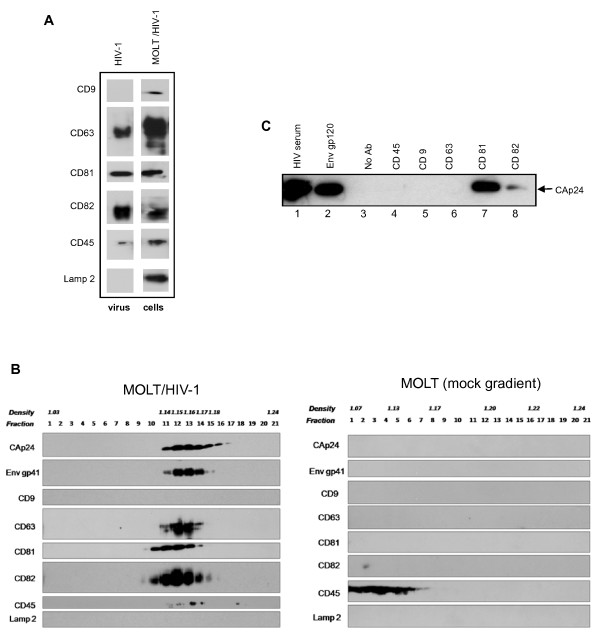
**The tetraspanins CD63, CD81 and CD82 are associated with purified HIV-1 virions**. (A). Cell lysate from MOLT/HIV-1 cells was run on SDS-PAGE and probed with antibodies against CD45, CD9, CD63, CD81, CD82, and Lamp2 as indicated ("cells"). Purified viral pellet from MOLT/HIV-1 was immunoblotted with the same antibodies ("virus"). (B). Purified virions produced by MOLT/HIV-1 cells (left panel) were loaded on 20–70% sucrose density gradient. After ultracentrifugation at equilibrium, the gradient was fractionated and the density (g/ml) of each fraction was determined, as indicated. Immunoblots of all fractions were performed using antibodies against Gag and Env, the tetraspanins CD63, CD81, CD82, or CD9, and CD45 or Lamp2 as controls. HIV-1 virions, as seen by the CAp24 and TMgp41, appeared in fractions with a density between 1.15–1.17 g/ml. In the same viral fractions, signals were obtained for the tetraspanins CD63, CD81 and CD82. Control gradient from uninfected MOLT cells is presented on the right panel. (C). Purified HIV-1 virions from MOLT/HIV-1 were submitted to immunoprecipitation with CD45, CD9, CD63, CD81 and CD82 antibodies (lane 4 to 7), or with HIV-1 serum and Env gp120 antibody as positive controls (lane 1 and 2) or without antibody as a negative control (No Ab – lane 3). Immunoprecipitated virions were run on SDS-PAGE gels and revealed with an anti-CAp24 antibody.

Particles produced by MOLT/HIV-1 cells were purified, and total viral proteins were analyzed for the presence of tetraspanins by immunoblotting (Fig. 4A). All proteins of interest were present in the cell lysates (Fig. 4A). However, we found that the tetraspanins CD81, CD63 and CD82 were present in the virus-containing pellet, while CD9 was not. The membrane protein Lamp2 was not found in the viral pellet, in agreement with the data obtained by immuno-confocal analysis. Only the cell surface marker CD45 was slightly detected in the virus-containing pellet (Fig. 4A).

To confirm the presence of tetraspanins in HIV-1 virions, we performed a more stringent purification by running the already purified virions on a sucrose density gradient (Fig. 4B) (see Materials and Methods). The fractions were analyzed for density, RT activity and relative amounts of different cellular membrane and viral proteins. RT activity (data not shown), and the CAp24 and TMgp41 proteins (Fig. 4B, lane 11–15) were found in fractions where HIV-1 virions are known to sediment (density of 1.15 – 1.18 g/ml) [38,39]. Large amounts of CD63, CD81 and CD82 co-sedimented with HIV-1 (Fig. 4B – left panel, lane 11–14), while CD9 and Lamp2 did not. In a mock gradient ("pellet" from uninfected MOLT cells, Fig. 4B – right panel), no signal was obtained for any of the tetraspanins indicating that they were not secreted from the cells in a pelletable form. Interestingly, CD81 and CD82 were also detected in a slightly lighter density fraction (lane 10), which could be due to their high cell surface expression and/or the potential contamination of viral particles by plasma membrane microdomains of a lighter density. In fact, contamination of HIV-1 sucrose density-equilibrium gradients with plasma membrane derived vesicles (microvesicles) has been previously observed [40-42]. CD45, which is an abundant cell surface protein was identified as a molecule that is highly expressed on microvesicles, but not found in HIV-1 virions [40]. The fact that CD45 was hardly detected (Fig. 4B, lane 12–14), suggests that these fractions contain only minimal amounts of microvesicles. We cannot completely exclude the presence of exosomes in the virus fractions because their range of sedimentation may vary from 1.08 to 1.22 g/ml [43] which overlaps with the HIV-1 virion density. However, CD9, which accumulates in exosomes [44,26,27], was not found in the gradient, indicating a minimal contamination (if any) of the purified virions by exosomes.

Thus, we observed that CD81, CD82, and CD63, were associated with HIV-1 virions produced by infected T-lymphoblastic cells.

### Anti-tetraspanin antibodies immunoprecipitate HIV-1 virions

As a complementary approach to document the incorporation of tetraspanins in HIV-1 virions, we examined whether anti-tetraspanin antibodies could immunoprecipitate purified HIV-1 virions. For this purpose, virus preparations were incubated with anti-tetraspanin antibodies coupled to Sepharose-G beads, and the viral content of the immunoprecipitated material was analyzed by immunoblotting (Fig. 4C). The anti-CAp24 immunoblot revealed that viral particles were immunoprecipitated from purified virus using an HIV-1 serum and anti-Env gp120 (Fig. 4C, lane 1 and 2), and anti-CD81 (lane 7) antibodies. A weak signal appeared using an anti-CD82 antibody (lane 8). No signal was detected in immunoprecipitates from the mock sample ("vesicles" of uninfected cells, data not shown), which indicates that the antibodies used for immunoprecipitation did not cross-react with the anti-CAp24 antibody. Non-specific immunoprecipitation of the virus was under the threshold of detection since no band was observed in the absence of antibody (lane 3), or with antibodies against CD45 (lane 4) or CD9 (lane 5).

Although CD63 was detected in the viral pellet (Fig. 4A and 4B), no CAp24 was detected in the CD63 immunoprecipitate (lane 6). We can speculate that even though CD63, CD81 and CD82 are associated with HIV-1 virions, only CD81 is well incorporated into the particle, which can be due to its tight interaction with HIV-1 proteins during assembly.

### HIV-1 Gag proteins form intracellular complexes with tetraspanins in MOLT/HIV-1 cells

**Figure 5:**
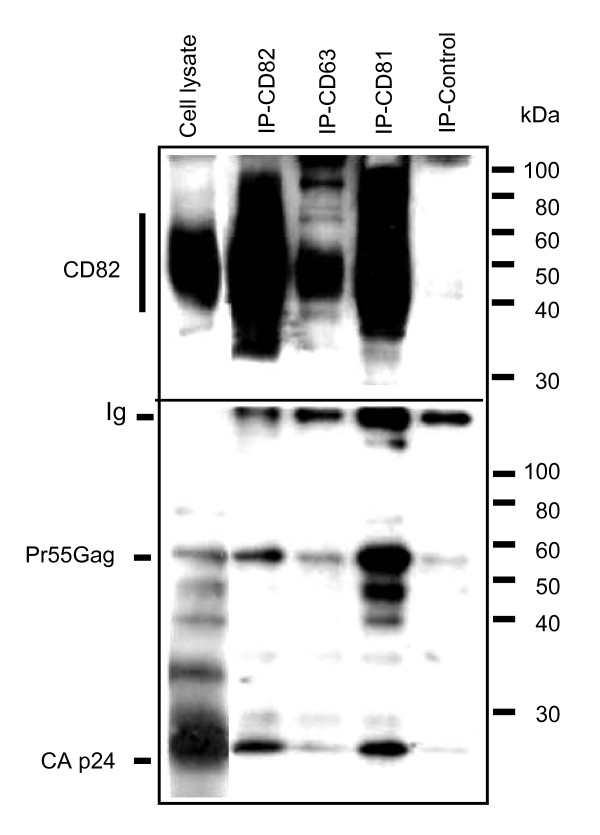
**HIV-1 Gag proteins form intracellular complexes with endogenous CD81 and CD82 tetraspanins**. Cell lysates from MOLT/HIV-1 cells were immunoprecipitated with antibodies directed against CD81, CD63 and CD82 or a control antibody. Non-immunoprecipitated (supernatants) or immunoprecipitated (IP) proteins were resolved by SDS-PAGE and blotted with an anti-HIV-1 human serum or an anti-CD82 antibody as indicated. The positions of Gag products and CD82 are indicated. The anti-CD82 blot shows the integrity of the tetraspanin web. The anti-HIV-1 blot shows intracellular Gag-tetraspanin interactions.

Since we found that CD81, and to a lesser extent CD82, were incorporated into newly made virions, we analyzed whether HIV-1 Gag proteins could associate with these tetraspanins in infected T cells (Fig. 5). MOLT/HIV-1 cells were lysed using a mild detergent and proteins were immunoprecipitated with antibodies directed against CD63, CD81, CD82 or with a control antibody. After SDS-PAGE under non-reducing conditions, membranes were blotted with an anti-CAp24 (bottom) or with an anti-CD82 (top) antibody. Figure 5 (bottom) shows that anti-CD81 antibodies clearly precipitated the Pr55Gag precursor and the mature CAp24, while background levels were detected with the control antibody and with anti-CD63. The Pr55Gag and CAp24 proteins were also slightly detected upon immuno-precipitation with anti-CD82. Blotting the same immunoprecipitates with an anti-CD82 antibody (Fig. 5, top) showed that the mild lysis conditions used maintained the tetraspanin web association since a significant amount of CD82 was recovered together with CD81 and CD63.

These data reveal that intracellular HIV-1 structural Gag proteins can strongly associate with CD81, and less with CD82, highlighting a potential role for CD81 in virus assembly. This result may also explain why we could not detect CD63, and very little CD82, in HIV-1 virions by immunoprecipitation (Fig. 4C).

### CD81 tetraspanin segregation from the cell surface impairs HIV-1 release

**Figure 6:**
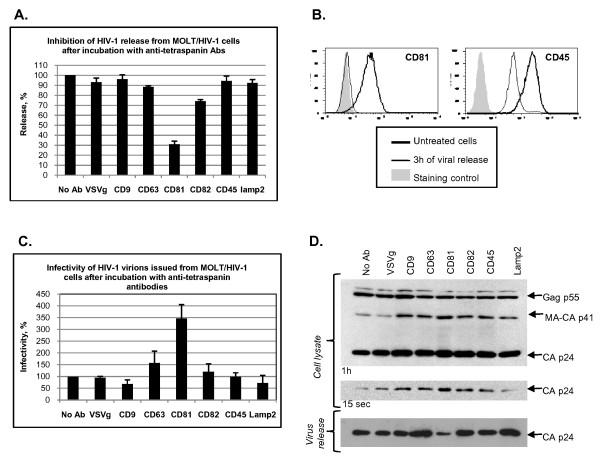
**Partial inhibition of HIV-1 release using anti-tetraspanin antibodies**. (A) MOLT/HIV-1 cells were incubated for one hour with anti-tetraspanin antibodies, or with anti-VSVg, anti-CD45 or anti-Lamp2; the antibodies were then removed, and virus release was measured in the supernatant 3 hours post incubation with the antibodies. The results of two independent experiments are presented on the chart. The percentage of virus release is evaluated by RT assay in comparison to the release in the absence of antibodies, normalized to 100%. (B) Cell surface tetraspanin inaccessibility after treatment of MOLT/HIV-1 cells with the anti-tetraspanin antibodies was evaluated by FACS analysis. The histograms present the surface staining of untreated cells and cells treated with anti-CD81 (first panel) and anti-CD45 (last panel), as indicated, at 3 hours post-viral release. When the proteins are expressed at the cell surface (i.e. CD81 or CD45), the antibody treatment leads to a decrease of the Mean Fluorescent Intensity measured. (C) Infectivity of the released virus after the treatment with anti-tetraspanin antibodies. The same amount of virus was inoculated on SupT1 cells, and the resulting RT activity from *de novo *produced virions was detected (See Materials and Methods). The infectivity obtained from the "No Ab" control virus was referred as 100%. (D) Virus maturation (and/or retention) as well as virus release at 3 hours after anti-tetraspanin treatment in MOLT/HIV-1 cells were evaluated by immunoblotting using an anti-CAp24 monoclonal antibody. Cells were lysed and 50 μg of proteins were deposited on a gel. On the upper panel ("cell lysate") two expositions of the film are presented: at 15 seconds, only the viral capsid could be detected; at 1 hour, all maturation products appeared. Partial inhibition of virus release could be observed on the lower panel ("virus release") which is consistent with that observed by the RT assay on Fig. 4A.

To evaluate the functional impact of Gag/tetraspanin interaction, we investigated the consequences of anti-tetraspanin antibody treatment on HIV-1 release. MOLT/HIV-1 cells were incubated with anti-tetraspanin antibodies for 1 hour, and virus release was monitored 3 hours post-treatment. This procedure can trigger either tetraspanin internalization [45] or fix tetraspanins on TEM at the cell surface and render them unable to function. We found that anti-CD81 antibodies decreased HIV-1 release by 3-fold (Fig. 6A). A lower effect was observed after cell treatment with anti-CD82, and practically no effect was seen with anti-CD9, anti-CD63 or with anti-VSVg, anti-Lamp2 or anti-CD45 control antibodies (Fig. 6A).

Flow cytometry analysis showed that one hour of incubation of MOLT/HIV-1 cells with anti-CD81 or anti-CD45 antibodies, even 3 hours after the treated cells were washed, caused a significant disappearance or inaccessibility of CD81 and CD45 from the cell surface (Fig. 6B). However, in contrast to the anti-CD81 results, anti-CD45 had no effect on virus release.

Following anti-CD81 treatment of the MOLT/HIV-1 producer cells, infectivity of those virions (normalized by RT activity) was about 2–3-fold higher as compared with virions from untreated MOLT/HIV-1 cells (Fig. 6C). Thus, the presence of CD81 on the virus could modulate its infectivity in cell culture in accordance with the recent data of Sato and collaborators (47) who reported a similar effect of other tetraspanins on HIV-1 infectivity.

Lastly, we examined Gag processing upon producer cell treatment with different anti-tetraspanin antibodies (Fig. 6D). We found that Gag maturation remained unchanged. In addition, there was retention of mature virions (as seen by the matured capsid CAp24) in producer cells treated with anti-CD81 consistent with the fact that there was less virus produced in the presence of an anti-CD81 antibody (Fig. 6D, lane CD81).

Taken together, these results suggest that an optimal HIV-1 particle production is dependent on the presence of functional or accessible CD81 in HIV-1 producing T lymphoblastic cells.

### CD81 tetraspanin silencing causes a partial inhibition of HIV-1 production and modulates virus infectivity

**Figure 7:**
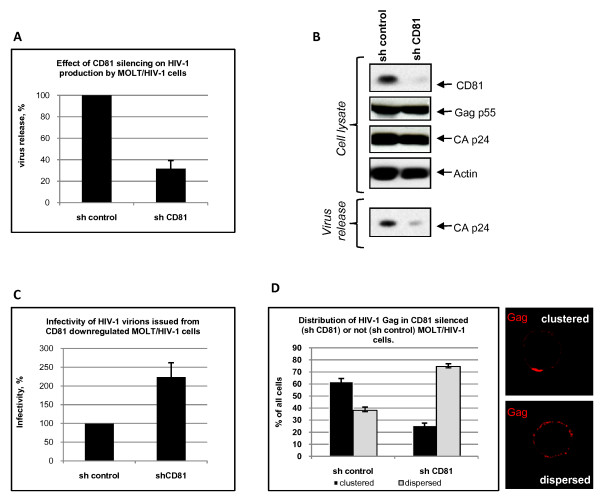
**Effects of CD81 downregulation by shRNA on viral production, HIV infectivity and Gag localization**. (A) Virus release was determined by measuring RT activity in the supernatant of both control and CD81 silenced MOLT/HIV-1 cells. It is expressed as a percentage of the control. Transduction of MOLT/HIV-1 cells with a CD81 shRNA led to an inhibition of HIV-1 production up to 70% (3-fold). (B) Immunoblots showing intracellular CD81 silencing and its effect on viral particle release. MOLT/HIV-1 cells were transduced by HIV-1 based lentivectors containing a shRNA against CD81 or a control shRNA. Three days later, the cells were washed, and resuspended in new medium for 6 hours to allow HIV-1 virion accumulation. The resulting viral particles were run on a SDS-PAGE gel and immunoblotted with an anti-CAp24 antibody to reveal virus particle release. The cells treated with the control shRNA (lane, "sh control") or with the anti-CD81 shRNA (lane "sh CD81") were lysed and total cell protein content were deposited on SDS-PAGE. Resulting immunoblots were probed with different antibodies as indicated. (C) Infectivity of virions issued from shRNA control or CD81 silenced MOLT/HIV-1. The same amount of virus was innoculated on SupT1 cells, and the resulting RT activity from *de novo *produced virions was detected (See Materials and Methods). The infectivity obtained from the shRNA control virus was referred as 100%. (D) Gag localization at the cell surface by immunofluorescence microscopy. After treatment with lentiviral vectors expressing shCD81 or control shRNA, MOLT/HIV-1 cells were fixed, permeabilized and stained for Gag (using anti-MAp17 antibody) as described in Materials and Methods. Two major phenotypes of Gag were observed: "clustered" – Gag is located in a cluster at one side of the cell surface; or "dispersed" – Gag is distributed all over the cell periphery as punctuated small dots. Patterns were quantified for CD81(-) cells and for the control cells; the numbers were reported on the chart.

To further investigate the effects of CD81 on HIV-1 production and infectivity, we silenced its expression in MOLT/HIV-1 cells using a lentivector (LV) expressing a shRNA directed against CD81. For this purpose, we transduced the cells with an HIV-1 based LV that encodes either a shRNA against CD81 or an irrelevant shRNA (control). Three days post LV transduction, the level of intracellular GFP expression reached 99% in both cell cultures showing a high level of cell transduction. We thus measured HIV-1 release in the cell supernatant by RT assay (Fig. 7A) and analysed the total cellular expression of Gag, CAp24 and CD81 by immunoblotting (Fig. 7B). Our results showed that in the CD81 shRNA transduced cells the level of expression of CD81 was three times lower than in the control cells (based on the mean fluorescence index as measured by FACS analysis – data not shown) and was barely detectable by immunoblotting (Fig. 7B). HIV-1 production by these cells was decreased by 70% (3-fold) as compared to cells transduced with the control LV-shRNA (Fig. 7A and 7B).

Virus production and CD81 silencing in the cells were analyzed by immunoblotting (Fig. 7B). Both virus release and CD81 level were strongly reduced in the shCD81 treated cells (Fig. 7B) showing that an efficient silencing of CD81 can lead to an important reduction of HIV-1 particle production. At the same time, LV infection of these cells did not have an effect on the intracellular expression of HIV-1 Gag or actin.

This result showed that the downregulation of CD81 by an interfering shRNA significantly impairs HIV-1 release.

Furthermore, the resulting virus issued from CD81 silenced MOLT/HIV-1 cells was tested for infectivity on SupT1 cells, as compared with the virus issued from shRNA control cells (Fig. 7C). Upon normalization of the virus by RT activity, we observed that HIV-1 produced by CD81 silenced cells was about 2.5 fold more infectious than the virus produced by control LV-treated MOLT/HIV-1 cells, suggesting that CD81 can modulate virus infectivity in cell culture.

### Downregulation of CD81 results in Gag redistribution at the cell surface

Down modulation of CD81 expression in MOLT/HIV-1 producer cells prompted us to examine by immunofluorescence microscopy Gag distribution in these cells ("sh CD81") and in control LV treated MOLT/HIV-1 cells ("sh control") (Fig. 7D). There are two major Gag distributions, namely in clusters at the cell surface ("clustered") and in a punctated form all over the cell periphery ("dispersed"). In CD81(+) cells, Gag appeared mainly clustered (~60% showed clustering and ~40% punctated), while in CD81(-) cells, Gag was mainly punctated (in more than 75% of the cells, Gag appeared in a punctated pattern at the cell surface).

This observation suggests that CD81 is required for the organization of HIV-1 Gag within functional TEMs which would favour HIV-1 assembly, release, and possibly transmission via the virological synapse [16,46].

## Discussion

This study was aimed at investigating the role of tetraspanins in the late steps of HIV-1 replication in chronically infected T cells (MOLT/HIV-1), one of the several chronically infected cell systems to study HIV-1 assembly [14]. In this work, we examined the putative association of HIV-1 with cellular tetraspanins such as CD9, CD63, CD81 and CD82, that can be found in endosomal and cell surface membranes of T cell lines. We found that HIV-1 Gag and Env structural proteins co-localized with these tetraspanins, but only CD63, CD81 and CD82 clustered at the cell surface together with the major viral structural proteins (Figs. 1, 2 and 3). These tetraspanins co-purified with HIV-1 virions, but only anti-CD81 and anti-CD82 antibodies were able to immunoprecipitate viral particles (Fig. 4). We were unable to immunoprecipitate HIV-1 virions with an anti-CD63 antibody in contrast to previous studies which reported anti-CD63 mediated immunoprecipitation of HIV-1 particles that were produced by macrophages (19, 20). In agreement with our data, Gag containing TEMs of MOLT/HIV-1 cells showed only a low level of CD63 (Fig. 5).

Next, we showed that intracellular Gag and capsid (CAp24) proteins were engaged in complexes with CD81, and much less with CD82, suggesting that HIV-1 proteins specifically interact, directly or indirectly, with CD81, which is part of a CD81/CD82/CD63 TEM in infected MOLT/HIV-1 cells (Fig. 5). This result is consistent with the fact that purified HIV-1 virions produced by these cells contain CD81, implying that CD81 might have a role during HIV-1 assembly in MOLT/HIV-1 cells. In agreement with this, incubation of MOLT/HIV-1 cells with an anti-CD81 antibody significantly impaired virus release (Fig. 6). Moreover, silencing CD81 expression by shRNA also resulted in a partial inhibition of viral particle production (Fig. 7).

Nyddeger and collaborators were the first to propose that tetraspanin-enriched microdomains (TEMs) can function as gateways for HIV-1 egress in HeLa cells [47]. This was also found to occur in macrophages [21] and in HIV-1 infected Jurkat T cells [16]. We previously reported that in MOLT/HIV-1 cells, viral particles and intracellular Gag can associate with the CD63 tetraspanin [14]. Our last findings showed that in these T cells, HIV-1 assembly can occur on TEMs (i.e. that Gag proteins use TEMs, composed at least of CD81/CD82/CD63), as a platform for particle assembly with a notable interaction, direct or indirect, between Gag and CD81.

CD82 could have been a good candidate for Gag assembly as it was found to co-localize with intracellular Gag and Env (Figs. 1, 2 and 3); however, an anti-CD82 antibody had a very moderate effect at immunoprecipitating intracellular Gag or inhibiting HIV-1 release. Either the targeted epitope of the anti-CD82 is less accessible or there is no major functional role for CD82 in virus formation and/or release, in comparison with CD81. Indeed, several monoclonal anti-tetraspanin antibodies were tested and were able to reproduce the results shown in Fig. 6 (data not shown). Thus only the effect of CD81 downregulation in MOLT/HIV-1 cells was investigated on HIV-1 release and infectivity. We showed that CD81 downregulation decreased virus production by 3-fold (Fig. 7A, B) and the resulting virus was more infectious (Fig. 7C), suggesting that CD81 tetraspanin incorporation is able to modulate HIV-1 infection, as this was recently proposed for HIV-1 infected CD4+ activated lymphocytes [48].

Thus one has to understand how CD81 segregation in TEM favors Gag assembly and release, and at the same time CD81 incorporation into virion renders viruses less infectious. In fact, the high concentration of Gag in TEM should certainly accelerate virus assembly at one cell pole (Fig. 7D), resulting in the favoring of HIV-1 transmission by cell-cell contacts via the virological synapse [16,46].

Tetraspanins, like other membrane cellular proteins present in the viral envelope [40], can tell us about the nature of the cellular compartment from which the virus buds or where HIV-1 accumulates. Nowadays, the site of HIV-1 assembly still remains controversial {i.e. mainly at the plasma membrane [16,18,22,49], and in late endosomes/MVB [30,10,17,23,20,11] or in plasma membrane invaginations [21]}. It appears that HIV-1 can accumulate in intracellular compartments not only during virus formation [14,17,20], but also after assembly at the cell surface followed by an endocytosis of Gag complexes [18,22]. This intracellular compartment where HIV-1 is targeted is enriched in specific tetraspanins that can vary from one cell to another. In fact, virus-containing compartment (VCC) was proposed to be enriched in (i) CD63 and MHC II [17,19,20] or (ii) CD9, CD53, CD81 and not CD63 [21] for monocyte derived primary macrophages (MDM); (iii) CD9 and CD81 [33] or (iv) CD63 and CD81 [50] for dendritic cells; and (v) CD63, CD81 and rarely CD9 [16] for T cells. In MOLT/HIV-1 cells, the virus-containing compartment seems to be enriched in CD81 and CD82. Therefore, it appears that a different set of tetraspanins can be recruited in tetraspanin-enriched VCC, where CD81 is always present. It was suggested that this compartment is different from the conventional endosomes, and its formation is induced by the virus [17]. What can differ between the VCC and the LE/MVB is the lack of acidification, permitting the persistence of infectious HIV-1 particles within cells for a long time [51,33,17]. The pH in VCC is mildly acidic due to the de-localization of the V-ATPase which is responsible for the acidification of the endosomal vesicles [17]. This might be due to the recruitment of specific tetraspanins within VCC which might counteract the V-ATPase subunits and consequently ensure an optimal environment for HIV-1 formation and/or storage. Indeed, it was shown that CD63 can associate with gastric H+, K+ ATPase and target it for degradation in lysosomes [52]. This could explain why we observed intracellular co-localization of CD63 with Gag and Env, but no effect from anti-CD63 on HIV release. Another explanation could be, as it was shown in previous work, that HIV infected T cells are resistant to inhibition of HIV-1 infection by a CD63 antibody, but sensitive to CD63 down regulation by siRNA [32]. Thus, CD63 could have a role in modulating the trafficking of these associated proteins and not play a direct role in HIV-1 assembly *per se*. Actually, there is some discrepancy on the role of CD63 in the late steps of HIV-1 replication, as Ruiz-Mateos et al. have shown that CD63 is not required for either the production or the infectivity of HIV-1 in MDM [31] while Chen and colleagues found the opposite [32].

Tetraspanins may serve as molecular facilitators, collecting proteins together [53], which suggests, in the context of HIV-1, that tetraspanins could facilitate virus assembly and release by bringing together Gag proteins and cellular factors, such as Tsg101, Alix, and others which are required at the budding site of HIV-1. Indeed, CD63 was found to participate in protein trafficking by inducing functional associations between protein complexes [53]. Consistent with these hypotheses, we observed the association of HIV-1 Gag and Env with CD81 and CD82. Our results showed that CD81 and CD82, and less CD63, are clustered with Gag in the VCC or at the plasma membrane. Thus, in reference to published views on the potential roles of cellular proteins in HIV-1 [54], either CD81/CD82 or CD63 are incorporated into virion particles simply because of their presence at the assembly site (Figs. 1, 2, 3 and 4), or because of their interaction with Gag (Fig. 5). Therefore, either these tetraspanins were swept up into the budding virions by this specific location (Fig. 4) but have no function, or they are purposefully incorporated to perform a function for the virus (Fig. 7). In the light of our results, only CD81 appeared to be functionally important for HIV-1 production in MOLT/HIV-1 cells (Fig. 7B). We may speculate that CD81, CD82 and CD63 are useful for the achievement of an optimal environment for HIV-1 assembly, while only CD81 interacts, directly or not, with the viral protein Gag and facilitates virus formation and egress. We report that there is an interaction between Gag (also CAp24) and the tetraspanin web within the infected cell (Fig. 5). It will be of interest to define the exact domain of HIV-1 Gag that interacts with CD81. In fact, in HTLV-1 (another human retrovirus), the MA domain of Gag associates with CD82 and CD81 inner loops [55].

Furthermore, we show a relationship between CD81 silencing in HIV-1 producing cells and Gag de-localization from the TEM which resulted in an inhibition of HIV-1 release. However, the downregulation of CD81 allowed the virus to be more infectious (Fig. 7C), suggesting that CD81 could also act as a cellular defense response against HIV-1 infection. Interestingly, the upregulation of CD81 mRNA synthesis was observed in primary CD4+ T lymphocytes infected by HIV-1 from patients [56]. Along this line, HIV-1 replication can be positively or negatively regulated through multiple interactions with host cell proteins, as we recently reported for the cellular human Disc Large scaffold protein (hDlg1) which restricts HIV-1 infectivity [57].

In conclusion, our study highlights the role of the CD81 tetraspanin in the late steps of HIV-1 replication in T lymphoblastic cells. Further investigations will be needed to reveal the sequential events that lead to TEM formation, cellular factor recruitments and the nature of the interaction between CD81 and the retroviral Gag proteins in the late steps of HIV-1 replication.

## Abbreviations

TEM: tetraspanin-enriched microdomains; PM: plasma membrane; HIV-1: human immunodeficiency virus type 1; MVB: multivesicular bodies; MA: matrix protein; CA: capsid protein; Env: envelope glycoproteins, LV: lentiviral vectors.

## Competing interests

The authors declare that they have no competing interests.

## Authors' contributions

BG carried out the virology work, confocal microscopy and quantification, lentiviral vectors assays and infections. VAA carried out virology, cell culture, viral immunoassays, lentivector production. FP participated in cell culture, infectious cell extracts for cellular immunoassays. MN carried out the cellular immunoassays of TEM. SW participated in immunoblots and confocal microscopy. CP, head group, participated in the writing work relative to Fig. 5. JLD, head group, participated writing the manuscript. HC, head group expert in tetraspanin biology, provided the tetraspanin antibodies and participated in the discussions and writing work. DM conceived the study, its design and coordination, and wrote the manuscript. All authors read and approved the final manuscript.
